# Maternal circulating metabolic biomarkers and their prediction performance for gestational diabetes mellitus related macrosomia

**DOI:** 10.1186/s12884-023-05440-9

**Published:** 2023-02-14

**Authors:** Yingdi Yuan, Qingyi Zhu, Xiaodie Yao, Zhonghua Shi, Juan Wen

**Affiliations:** 1 Department of Pediatrics, The First People’s Hospital of Lianyungang, Xuzhou Medical University Affiliated Hospital of Lianyungang (Lianyungang Clinical College of Nanjing Medical University), Lianyungang, China; 2 Nanjing Maternity and Child Health Care Institute, Women’s Hospital of Nanjing Medical University, Nanjing Maternity and Child Health Care Hospital, Nanjing, China; 3 Department of Obstetrics, Women’s Hospital of Nanjing Medical University, Nanjing Maternity and Child Health Care Hospital, Nanjing, China

**Keywords:** Gestational diabetes mellitus, Macrosomia, Metabolic biomarkers, Predictive model, ROC curve

## Abstract

**Introduction:**

Gestational diabetes mellitus (GDM), a metabolism-related pregnancy complication, is significantly associated with an increased risk of macrosomia. We hypothesized that maternal circulating metabolic biomarkers differed between women with GDM and macrosomia (GDM-M) and women with GDM and normal neonatal weight (GDM-N), and had good prediction performance for GDM-M.

**Methods:**

Plasma samples from 44 GDM-M and 44 GDM-N were analyzed using Olink Proseek multiplex metabolism assay targeting 92 biomarkers. Combined different clinical characteristics and Olink markers, LASSO regression was used to optimize variable selection, and Logistic regression was applied to build a predictive model. Nomogram was developed based on the selected variables visually. Receiver operating characteristic (ROC) curve, calibration plot, and clinical impact curve were used to validate the model.

**Results:**

We found 4 metabolism-related biomarkers differing between groups [CLUL1 (Clusterin-like protein 1), VCAN (Versican core protein), FCRL1 (Fc receptor-like protein 1), RNASE3 (Eosinophil cationic protein), FDR <  0.05]. Based on the different clinical characteristics and Olink markers, a total of nine predictors, namely pre-pregnancy body mass index (BMI), weight gain at 24 gestational weeks (gw), parity, oral glucose tolerance test (OGTT) 2 h glucose at 24 gw, high-density lipoprotein (HDL) and low-density lipoprotein (LDL) at 24 gw, and plasma expression of CLUL1, VCAN and RNASE3 at 24 gw, were identified by LASSO regression. The model constructed using these 9 predictors displayed good prediction performance for GDM-M, with an area under the ROC of 0.970 (sensitivity = 0.955, specificity = 0.886), and was well calibrated (*P*_Hosmer-Lemeshow test_ = 0.897).

**Conclusion:**

The Model included pre-pregnancy BMI, weight gain at 24 gw, parity, OGTT 2 h glucose at 24 gw, HDL and LDL at 24 gw, and plasma expression of CLUL1, VCAN and RNASE3 at 24 gw had good prediction performance for predicting macrosomia in women with GDM.

**Supplementary Information:**

The online version contains supplementary material available at 10.1186/s12884-023-05440-9.

## Introduction

Gestational diabetes mellitus (GDM) is a metabolism-related pregnancy complication, defined as any degree of glucose intolerance with onset or first recognition during pregnancy [1]. The International Diabetes Federation estimated that 16.7% (21.1 million) of live births to women in 2021 had hyperglycemia during pregnancy [2]. Of these, more than 80% were due to GDM [2]. In recent years, with the improvement of China’s living standards and the implementation of the “universal two-child” policy, there are more and more overweight or elderly pregnant women, resulting in a sharp increase in the incidence of GDM in China [3]. Numerous studies have shown that women with GDM have a significantly increased risk of having a baby with macrosomia [4]. The GDM related macrosomia not only significantly increases short-term maternal and infant complications such as shoulder dystocia, postpartum hemorrhage and neonatal asphyxia, but also greatly increases the risk of long-term obesity, diabetes and metabolic syndrome [4]. And the DOHaD theory holds that the nutritional and nurturing environment in the first 1000 days of life is crucial to an individual’s health throughout life [5]. Therefore, early screening of GDM related macrosomia is of great significance not only for clinical control of macrosomia, but also for reducing maternal and infant complications and delaying the development of obesity or diabetes in the offspring of women with GDM.

The mechanism of the association between GDM and macrosomia is not fully understood. Risk factors including older age, overweight and obesity, excessive weight gain during pregnancy, hyperglycemia and hyperlipidemia in women developing GDM, do only partly explain the association between GDM and macrosomia risk [4], indicating that GDM may promote or induce additional maternal metabolic changes. Women with GDM have been shown to present significant metabolic changes, and studies have measured levels of a single or a few metabolic biomarkers in GDM [6–9], some of which were related to newborn weight status [7, 10]. For example, early pregnancy leptin and TNFα were reported as determinants of birth weight in women with normal weight [10], and plasma-glycated CD59 was indicated as a simple and accurate biomarker for detection of GDM in early pregnancy and risk assessment of delivering a large for gestational age (LGA) infant [7]. Differences in circulating metabolic biomarkers between GDM women with and without macrosomia could reflect macrosomia specific metabolic changes, reflecting different macrosomia risks. This biomarker phenotyping could be used to target women with GDM at the highest risk of macrosomia, to tailor GDM follow-up and preventive measures.

Overall, as women with GDM have a higher risk of having a baby with macrosomia, there should be systematic screening for macrosomia to increase early detection. The Proximity Extension Assay (PEA) technology combines the advantages of antibody-based and DNA-based methods to provide a unique tool for protein biomarker discovery and development [11]. This is a scalable, multiplex, and highly specific method for quantifying the concentrations of hundreds of protein biomarkers simultaneously [11]. In this study, using the PEA technology, we assessed whether a multiplex panel of 92 circulating metabolic biomarkers differed between GDM women with and without macrosomia. Furthermore, based on the different circulating metabolic markers and clinical characteristics, we analysed and identified risk factors affecting risk of GDM related macrosomia, and developed a scientific risk prediction model for obstetricians to conduct early screening of GDM related macrosomia.

## Methods

### Participants and sample collection

This was a retrospective, electronic medical records-based, case-control study. Participants were recruited at the Women’s Hospital of Nanjing Medical University from Jan 2020 to June 2020. The Ethics Committee of Women’s Hospital of Nanjing Medical University approved the study, and all participants signed informed consent. GDM was diagnosed by 75 g oral glucose tolerance test (OGTT) performed at 24 gestational weeks (fasting blood glucose ≥5.1 mmol/L, 1 h blood glucose ≥10.0 mmol/L, or 2 h blood glucose ≥8.5 mmol/L) [12], and macrosomia was defined as birth weight more than 4000 g. The pregnant women with GDM and macrosomia (birth weight ≥ 4000 g, GDM-M) were assigned to the case group, while women with GDM and normal neonatal weight (2500 g ≤ birth weight < 4000 g, GDM-N) were assigned to the control group. Participants diagnosed with diabetes before pregnancy or other pregnancy complications were excluded from this study. A total of 88 women with GDM were recruited in the study, including 44 GDM-M and 44 GDM-N. They were unrelated ethnic Han Chinese. All women with GDM were classified as GDM A1, which is controlled with diet and exercise, without the use of medications. Fasting (≥ 6 hours) blood samples from the 88 women with GDM were collected on the day of OGTT at 24 gestational weeks (gw), and EDTA plasma was stored at − 80 °C until analysis.

We also collected multiple obstetric data through chart review, including general maternal features (age, height, pre-pregnancy weight, gravidity, and parity), pregnancy information at 24 gw [weight, fasting glucose, OGTT 1 h glucose, OGTT 2 h glucose, glycosylated hemoglobin (HbA1c), total cholesterol (TC), triglyceride (TG), high-density lipoprotein (HDL), low-density lipoprotein (LDL)], fetal features (sex), and some maternal and neonatal outcomes [hospital stays, intrapartum weight, premature rupture of membrane, fetal distress, polyhydramnios (> 2000 ml in the third trimester), delivery mode (spontaneous labor or cesarean section), intrapartum fever (intrapartum temperature > 38 °C), gestational weeks at birth, birth weight, Apgar score, postpartum hemorrhage (measured blood loss ≥500 ml), postpartum fasting blood glucose, postpartum 2 h postprandial blood glucose, and postpartum hemoglobin] (Tables 1 and 2). Pre-pregnancy body mass index (BMI, kg/m^2^) was calculated as maternal pre-pregnancy weight divided by the square of height. Parity did not include this pregnancy and was divided into 0 (primiparas) and ≥ 1 (multiparas).

**Table 1:** Maternal and neonatal outcomes

Variables	GDM-M (***n*** = 44)	GDM-N (***n*** = 44)	***P***
Hospital stays (days)	6.2 ± 2.0	4.6 ± 1.3	< 0.001
Weight gain during pregnancy (kg)	13.5 ± 4.9	10.0 ± 3.2	< 0.001
Premature rupture of membrane	8 (18.2%)	0	0.009
Fetal distress	2 (4.5%)	0	0.474
Polyhydramnios	4 (9.1%)	0	0.125
Cesarean delivery	25 (56.8%)	14 (31.8%)	0.018
Intrapartum fever	4 (9.1%)	5 (11.4%)	1.000
Gestational weeks at birth	39.5 ± 0.9	39.0 ± 0.9	0.016
Birth weight (g)	4162.7 ± 141.7	3224.1 ± 301.7	< 0.001
1-min Apgar score < 10	1 (2.3%)	0	1.000
Postpartum hemorrhage	1 (2.3%)	1 (2.3%)	1.000
Postpartum fasting blood glucose (mmol/L)	4.9 ± 0.7	4.4 ± 0.5	0.016
Postpartum 2 h postprandial blood glucose (mmol/L)	5.8 ± 0.7	6.0 ± 1.6	0.539
Postpartum hemoglobin (g/L)	117.7 ± 14.9	124.1 ± 10.1	0.021

*GDM-M* women with gestational diabetes mellitus and macrosomia, *GDM-N* women with gestational diabetes mellitus and normal neonatal weight

**Table 2:** Characteristics of the pregnant women

Variables	GDM-M (***n*** = 44)	GDM-N (***n*** = 44)	***P***
age (years)	30.5 ± 3.4	29.7 ± 2.9	0.239
Height (cm)	164.5 ± 3.9	162.1 ± 5.1	0.013
Pre-pregnancy weight (kg)	54.5 ± 5.3	47.9 ± 4.7	< 0.001
Pre-pregnancy BMI (kg/m^2^)	20.1 ± 1.6	18.2 ± 1.5	< 0.001
Weight gain at 24 gw (kg)	7.4 ± 3.5	5.3 ± 2.7	0.002
Gravidity	2 (1, 3)	1 (1, 2)	0.036
Parity (0, 1)	26, 18	36, 8	0.019
Fetal sex (boy, girl)	30, 14	24, 20	0.189
Fasting glucose at 24 gw (mmol/L)	5.0 ± 0.5	4.7 ± 0.5	0.016
OGTT 1 h glucose at 24 gw (mmol/L)	9.7 ± 0.9	9.3 ± 1.2	0.066
OGTT 2 h glucose at 24 gw (mmol/L)	8.6 ± 0.7	8.1 ± 1.0	0.010
HbA1c at 24 gw (%)	5.2 ± 0.5	5.1 ± 0.3	0.114
TC at 24 gw (mmol/L)	5.9 ± 1.0	5.9 ± 0.8	0.799
TG at 24 gw (mmol/L)	2.5 ± 1.8	2.2 ± 1.0	0.279
HDL at 24 gw (mmol/L)	2.3 ± 0.4	2.6 ± 0.3	< 0.001
LDL at 24 gw (mmol/L)	3.2 ± 0.8	2.8 ± 0.6	0.018

*GDM-M* women with gestational diabetes mellitus and macrosomia, *GDM-N* women with gestational diabetes mellitus and normal neonatal weight, *BMI* Body mass index; gw, gestational weeks, *OGTT* oral glucose tolerance test, *HbA1c* glycosylated hemoglobin, *TC* total cholesterol, *TG* triglyceride, *HDL* high-density lipoprotein, *LDL* low-density lipoprotein

### Multiplex biomarker analysis

EDTA plasma samples from 44 GDM-M and 44 GDM-N were determined using the Olink Proseek multiplex metabolism panel at the Shanghai Biotechnology Corporation, Shanghai, China, that had developed the PEA technology. This panel includes 92 proteins that are either established biomarkers or exploratory proteins with a high potential as new metabolic disease biomarkers. More detailed information can be found at the Olink website (https://olink.com/products-services/target/biological-process/). The assay protocols are available online and can be viewed using the link (https://olink.com/content/uploads/2022/05/olink-target-96-short-instructions.pdf). The obtained results are reported as Ct Normalized Protein expression (NPX) values. NPX values are obtained by normalizing Ct-values against extension control, inter-plate control and a correction factor. NPX values are on log2scale where a high NPX value corresponds to a high protein concentration and can be linearized by using the formula 2^NPX. Values below the lowest level of detection for each marker, based on the negative controls analyzed in each run, were set to LOD (limit of detection) value. Acronyms/full names and analytical details of the 92 Olink metabolism biomarkers are presented in Table S1. The raw data for the Olink Proseek multiplex metabolism assay is shown in Table S2.

### Statistical analyses

Statistical analyses were performed using SPSS v25.0 and R software (version 4.1.3). Missing values were imputed using the Random Forests algorithm. Continuous variables were described as mean (SD) when normally distributed and median (25th, 75th) when skewed distributed, and compared between the two study groups by Student’s *t*-test or non-parametric Mann-Whitney test, while categorical variables were displayed as frequency (percentage) and compared by *χ*^2^ test or Fisher exact test. For Olink markers, Student’s *t*-test adjusting for multiple testing (false discovery rate, FDR) was used to identify significantly different markers between the two study groups.

Based on the significantly different clinical characteristics (*P* <  0.05) and Olink markers (FDR <  0.05), we sought to build predictive models for GDM-M at 24 gw. First, the R *glmnet* package was used to run LASSO (least absolute shrinkage and selection operator) regression to select the optimal predictors of the present risk factors [13]. As the dependent variable is whether macrosomia is present or not, we set family = “binomial” and set type.measure = “deviance”. Based on the binomial family and the type measure of deviance, the LASSO regression analysis runs a ten-fold cross-validation for centralization and normalization of the included variables and then picks the best lambda value. *Lambda.1se* gives a model with good performance but the least number of independent variables. Then the R *rms* package was used to run logistic regression to construct a predictive model for GDM-M by introducing the factors selected in the LASSO regression. All of the selected factors were applied to develop nomogram prediction model. A graphical nomogram was also produced for the constructed model so that the individual-specific risk of macrosomia in women with GDM could be easily approximated [14].

Further, several kinds of validation methods were used to estimate the accuracy of the risk prediction model [13, 15]. The R *pROC* package was used to construct receiver-operator characteristic (ROC) curve, and the area under the ROC curve (AUC) was used to provide good discrimination for the quality of the risk nomogram to separate true positives from false positives. The sensitivity, specificity, negative predictive value (NPV) and positive predictive value (PPV) were calculated to illustrate the model effects using the “best threshold” criteria of the ROC curve. The R *rms* package was used to draw and calculate the calibration curves, which were used to evaluate the calibration of the GDM-M risk nomogram, accompanied by the Hosmer-Lemeshow test. And the R *rmda* package was used to draw clinical impact curves to determine the clinical practicability of the GDM-M prediction nomogram. The reported statistical significance levels were all two-sided, with *P* <  0.05 considered significant.

## Results

### Participants characteristics

This study included 88 women with GDM, including 44 GDM-M and 44 GDM-N. The maternal and neonatal outcomes in the GDM-M and GDM-N groups are shown in Table 1. The hospital stays, weight gain during pregnancy, gestational weeks at birth, birth weight, and postpartum fasting blood glucose in the GDM-M group were significantly higher than those in GDM-N group (6.2 days vs. 4.6 days, 13.5 kg vs. 10.0 kg, 39.5 weeks vs. 39.0 weeks, 4162.7 g vs. 3224.1 g, and 4.9 mmol/L vs. 4.4 mmol/L, respectively, *P* <  0.05), and the postpartum hemoglobin in the GDM-M group was significantly lower than that in GDM-N group (117.7 g/L vs. 124.1 g/L, *P* = 0.021). The rates of premature rupture of membrane and cesarean delivery were also higher in the GDM-M group than in the GDM-N group (18.2% vs. 0, and 56.8% vs. 31.8%, respectively, *P* <  0.05). Fetal distress, polyhydramnios, intrapartum fever, Apgar score, postpartum hemorrhage and postpartum 2 h postprandial blood glucose were similar between the 2 study groups (*P* > 0.05).

Then we retrospectively analysed the characteristics of pregnant women with GDM at 24 gw (Table 2). The height, pre-pregnancy weight, pre-pregnancy BMI, weight gain, gravidity, fasting glucose, OGTT 2 h glucose, and LDL in the GDM-M group were significantly higher than those in GDM-N group (164.5 cm vs. 162.1 cm, 54.5 kg vs. 47.9 kg, 20.1 kg/m^2^ vs. 18.2 kg/m^2^, 7.4 kg vs. 5.3 kg, 2 vs. 1, 5.0 mmol/L vs. 4.7 mmol/L, 8.6 mmol/L vs. 8.1 mmol/L, and 3.2 mmol/L vs. 2.8 mmol/L, respectively, *P* <  0.05), and the HDL in the GDM-M group was significantly lower than that in GDM-N group (2.3 mmol/L vs. 2.6 mmol/L, *P* < 0.001). For parity, the rate of multiparas (parity = 1) was higher in the GDM-M group than in the GDM-N group (40.9% vs. 18.2%, *P* = 0.019). Maternal age, fetal sex, OGTT 1 h glucose, HbA1c, TC and TG were similar between the 2 study groups (*P* > 0.05).

### Olink biomarker analysis

**Fig. 1:**
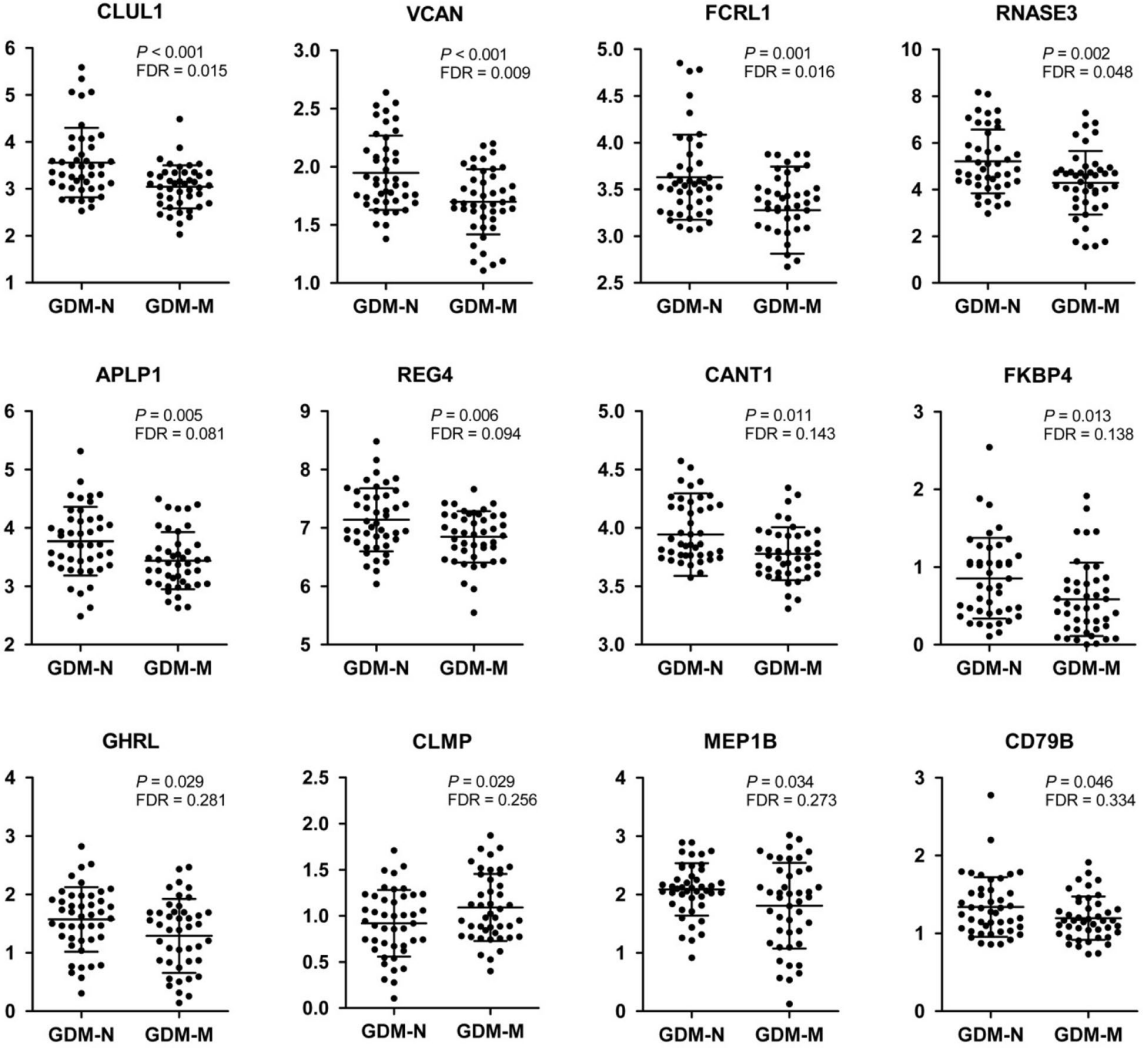
Individual levels of the 12 circulating Olink metabolism biomarkers differed significantly between women with gestational diabetes mellitus and normal neonatal weight (GDM-N) and women with gestational diabetes mellitus and macrosomia (GDM-M). Data are given as normalized protein expression (NPX). Horizontal bars represent mean ± SD. CLUL1, Clusterin-like protein 1; VCAN, Versican core protein; FCRL1, Fc receptor-like protein 1; RNASE3, Eosinophil cationic protein; APLP1, Amyloid-like protein 1; REG4, Regenerating islet-derived protein 4; CANT1, Soluble calcium-activated nucleotidase 1; FKBP4, Peptidyl-prolyl cis-trans isomerase FKBP4; GHRL, Appetite-regulating hormone; CLMP, CXADR-like membrane protein; MEP1B, Meprin A subunit beta; CD79B, B-cell antigen receptor complex-associated protein beta chain

Among the 92 Olink biomarkers, 88 were satisfactorily identified in the pregnant women. AHCY (Adenosyl homocysteinase), DIABLO (Diablo homolog, mitochondrial), DAB2 (Disabled homolog 2), and TSHB (Thyrotropin subunit beta) were excluded from further analysis as the 2 formers were not detected in measurable amounts. DAB2 and TSHB were only measurable in about half of the samples. We identified 12 of the 88 detected biomarkers as significantly different between GDM-M and GDM-N (*P* < 0.05, Table S1 and Fig. 1). Eleven biomarkers were significantly lower in GDM-M group [CLUL1 (Clusterin-like protein 1), VCAN (Versican core protein), FCRL1 (Fc receptor-like protein 1), RNASE3 (Eosinophil cationic protein), APLP1 (Amyloid-like protein 1), REG4 (Regenerating islet-derived protein 4), CANT1 (Soluble calcium-activated nucleotidase 1), FKBP4 (Peptidyl-prolyl cis-trans isomerase FKBP4), GHRL (Appetite-regulating hormone), MEP1B (Meprin A subunit beta), CD79B (B-cell antigen receptor complex-associated protein beta chain)], and CLMP (CXADR-like membrane protein) was significantly higher in GDM-M group. After adjusting for multiple testing, CLUL1, VCAN, FCRL1 and RNASE3 still differed significantly between the two study groups (FDR < 0.05).

### Predictive model construction

**Fig. 2:**
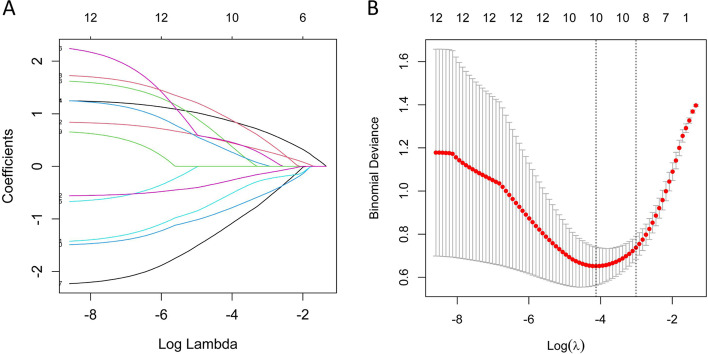
Variable selection by the LASSO binary logistic regression model. **A** A coefficient profile plot was constructed against the log(lambda) sequence. Eleven variables with nonzero coefficients were selected by deriving the optimal lambda. **B** Following verification of the optimal lambda in the LASSO model, we plotted the binomial deviance curve versus log(lambda) and drew dotted vertical lines based on 1 standard error criteria. LASSO, Least absolute shrinkage and selection operator

**Fig. 3:**
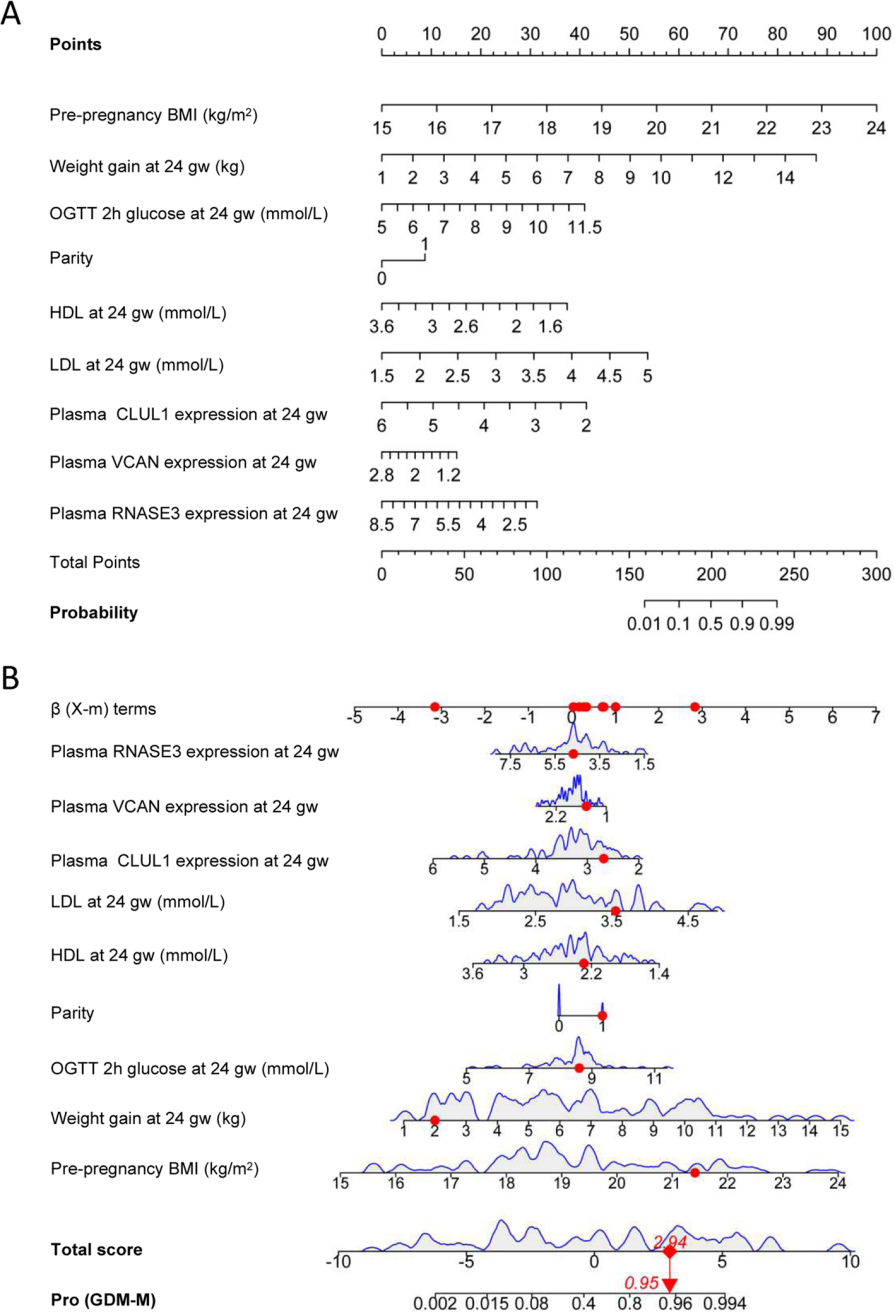
GDM-M risk nomogram prediction model. **A** Risk factors of pre-pregnancy BMI, weight gain at 24 gw, OGTT 2 h glucose at 24 gw, parity, HDL at 24 gw, LDL at 24 gw, plasma CLUL1 expression at 24 gw, plasma VCAN expression at 24 gw and plasma RNASE3 expression at 24 gw for nomogram prediction model. **B** Dynamic nomogram used as an example. GDM-M, women with gestational diabetes mellitus and macrosomia; BMI, body mass index; gw, gestational weeks; OGTT, oral glucose tolerance test; HDL, high-density lipoprotein; LDL, low-density lipoprotein; CLUL1, Clusterin-like protein 1; VCAN, Versican core protein; RNASE3, Eosinophil cationic protein

LASSO regression analysis was used to select predictive variables from the significantly different clinical characteristics [pre-pregnancy BMI, weight gain at 24 gw, gravidity, parity, fasting glucose at 24 gw, OGTT 2 h glucose at 24 gw, HDL at 24 gw and LDL at 24 gw] and Olink biomarkers (plasma expression of CLUL1, VCAN, FCRL1 and RNASE3 at 24 gw), and logistic regression was used to establish the predictive model. Nine of the original 12 variables were included in the predictive model, namely pre-pregnancy BMI, weight gain at 24 gw, parity, OGTT 2 h glucose at 24 gw, HDL and LDL at 24 gw, and plasma expression of CLUL1, VCAN and RNASE3 at 24 gw, as predictors (Fig. 2). These 9 variables had nonzero coefficients in the LASSO regression model. The predictive model was presented as a nomogram, which was used to quantitatively predict the risk probability of macrosomia in women with GDM (Fig. 3A). For example, using the nomogram model, a GDM woman with pre-pregnancy BMI of 21.41 kg/m^2^, weight gain of 2 kg, OGTT 2 h glucose of 8.6 mmol/L, HDL of 2.29 mmol/L, LDL of 3.55 mmol/L, CLUL1 expression of 2.68 (NPX value), VCAN expression of 1.48, and RNASE3 expression of 4.68 at 24 gw, has an estimated probability of macrosomia of 0.95 (Fig. 3B).

### Predictive model validation

**Fig. 4:**
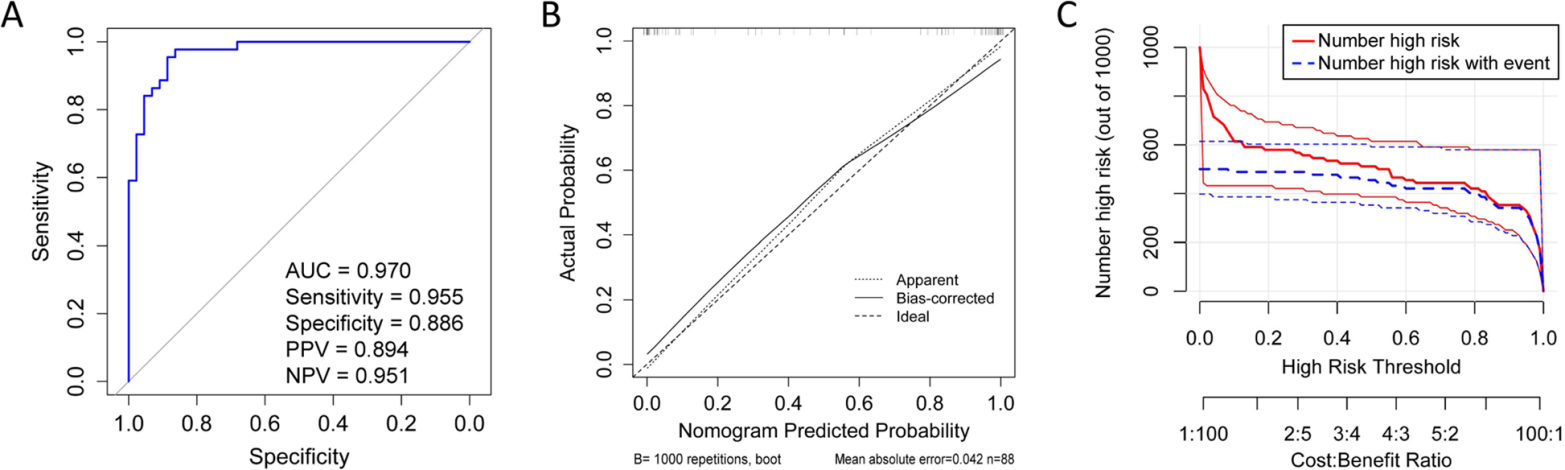
Validation of the GDM-M predictive model. **A** Receiver operating characteristic curve (ROC) validation of the GDM-M risk nomogram prediction. The y-axis represents the true positive rate of the risk prediction; the x-axis represents the false positive rate of the risk prediction; **B** Calibration curves of the predictive GDM-M risk nomogram. The y-axis represents actual diagnosed cases of GDM-M, the x-axis represents the predicted risk of GDM-M. The diagonal dotted line represents a perfect prediction by an ideal model, the solid line represents the performance of the GDM-M risk nomogram, with the results indicating that a closer fit to the diagonal dotted line represents a better prediction; **C** The clinical impact curve for the GDM-M predictive model. Of 1000 patients, the red solid line shows the total number who would be deemed high risk for each risk threshold. The blue dashed line shows how many of those would be true positives (cases). GDM-M, women with gestational diabetes mellitus and macrosomia. AUC, Area under the ROC curve; PPV, positive predictive value; NPV, negative predictive value

The ROC curve was used to evaluate the discriminatory capacity of the predictive model. For the predictive model, the pooled AUC of the nomogram is 0.970 (sensitivity = 0.955, specificity = 0.886, PPV = 0.894, and NPV = 0.951), which indicates good performance (Fig. 4A). To calibrate the predictive model, we then used a calibration plot and Hosmer-Lemeshow test. Based on the calibration curves (Fig. 4B), the predictive model fitted the data very well. The Hosmer-Lemeshow test showed the predicted and actual probability were highly consistent (*P* = 0.897). In the clinical impact curves for the predictive model (Fig. 4C), the red solid line shows the total number who were deemed high risk for each risk threshold of 1000 patients, and the blue dashed line shows how many of those were true positives. We also compared the discriminatory capacity between models with and without the Olink biomarkers. The AUC of the model included clinical characteristics and Olink biomarkers (AUC = 0.970) was higher than the AUC of the model just included clinical characteristics (AUC = 0.937), but the difference between models was not significant (Z = 1.43, *P* = 0.153).

## Discussion

The pathogenesis of macrosomia induced by GDM is particularly complex, involving many factors such as heredity, nutrition, and metabolic disturbance [4, 16, 17]. Recent studies have shown that the high glucose environment in the mother of GDM can stimulate the expression of various molecules (such as PTH-rP, PTH-R1, VEGF, CD31, etc.) from the placenta to the fetus, and combine with the high glucose in the uterus, resulting in fetal overgrowth [18, 19]. In our previous study, we reported that the expressions of GDF3, PROM1, AC006064.4, lnc-HPS6–1:1 and circ_0014635 were significantly increased, and the expression of lnc-ZFHX3–7:1 was significantly decreased in cord blood exosomes of the women with GDM-M [20]. Even so, current studies cannot fully elucidate the molecular mechanism of GDM-induced macrosomia [21], suggesting that there may be other mechanisms involved in the maternal and fetal communication stimulated by maternal high glucose.

In this retrospective study of women with GDM, we found a dysregulation of maternal circulating metabolic biomarkers in pregnancy with GDM-M by using Olink multiplex proteomics. Among the 92 metabolism linked biomarkers, we identified 4 markers different for women with GDM-M compared with women with GDM-N after adjusting for multiple testing, namely CLUL1, VCAN, FCRL1 and RNASE3. We are unaware of previous studies of pregnancy cohorts using this Olink metabolism linked multiplex biomarkers analysis. Several previous studies of pregnancy cohorts using Olink cardiovascular I/II or inflammation panel, have found maternal circulating Olink biomarkers differed by preeclampsia subtypes [22], associated with cord blood leukocyte telomere length [23], and in relation to abdominal fat distribution [24].

The dysregulated metabolic biomarkers identified in the GDM-M group in this study (CLUL1, VCAN, FCRL1 and RNASE3) may reflect heterogeneous physiological and pathological processes linked to the fetal development and metabolism. Although we found no previous studies investigating prospective associations of the dysregulated markers with GDM related macrosomia, all have been implicated in metabolic bioprocess in other settings. CLUL1 was firstly reported as a novel retinal specific clusterin (CLU)-like protein [25]. A BLAST search showed the CLUL1 and CLU proteins have a 23 to 25% sequence identity [25]. CLU is a highly conserved secreted glycoprotein widely expressed in many tissues [26], which has been suggested to function as a molecular chaperone [27]. Recently, it is reported that plasma CLU alteration could induce HDL dysfunction and contribute to peripheral artery disease that is aggravated by type 2 diabetes (T2D) [28]. VCAN, a large chondroitin sulfate proteoglycan in the extracellular matrix, could facilitate chondrocyte differentiation and regulate joint morphogenesis, having an important role in the musculoskeletal health [29]. In a large meta-analysis of genome-wide association studies for lean mass, *VCAN* loci (rs2287926) was identified and successfully replicated for whole body lean mass, and was important for sarcopenia diagnosis [30]. Moreover, a significant correlation between the *VCAN* loci and body fat mass in non-elderly individuals with T2D was also successfully replicated in another cross-sectional study [31]. FCRL1, a newly identified co-receptor of B cell receptors (BCR), was passively recruited into B cell immunological synapses upon BCR engagement in the absence of FCRL1 cross-linking, thus regulating B cell activation and function [32]. In a genome-wide association analysis of autoantibody positivity in type 1 diabetes (T1D) cases, the authors associated rs4971154 in exon 5 of *FCRL1* with islet antigen-2 (IA-2A), one of T1D-associated anti-islet autoantibodies [33]. RNASE3, a member of the RNaseA superfamily involved in host immunity, is expressed by leukocytes and shows broad-spectrum antimicrobial activity [34]. Together with a direct antimicrobial action, RNASE3 exhibits immunomodulatory properties [34]. The roles of FCRL1 and RNASE3 in inflammatory pathways point to an important role of the immune system in metabolic pathology in GDM related macrosomia. Whether these biomarkers might serve as treatment targets remains to be assessed in future studies.

By combining the above Olink biomarkers and different clinical characteristics, a total of 9 predictors, namely pre-pregnancy BMI, weight gain at 24 gw, parity, OGTT 2 h glucose at 24 gw, HDL and LDL at 24 gw, and plasma expression of CLUL1, VCAN and RNASE3 at 24 gw, were identified by LASSO regression. The model constructed using these 9 predictors displayed good prediction performance for GDM-M, with an AUC of 0.970, and was well calibrated. In the present study, we used a novel statistical method (LASSO regression) to identify the risk factors for GDM-M. The LASSO regression analysis minimizes prediction error for a quantitative response variable by imposing a constraint on the model parameters that cause the regression coefficients for some variables to shrink toward zero [13], thus providing more accurate results. A graphical nomogram was produced for obstetricians to easily use the constructed model to quantitatively predict the risk probability of macrosomia in women with GDM. Moreover, ROC, calibration and clinical impact curves were constructed to verify the accuracy and stability of the model.

However, our study had some limitations. First, this is a retrospective study. Second, this study was limited by funding, and we were unable to conduct Olink biomarker analysis for more women with GDM. Therefore, we could not divide the limited sample into training set and validation set. And the population we studied was limited to ethnic Han Chinese in Nanjing city, so caution should be taken when generalizing our findings to other populations. Third, the present study does lack a review of other factors contributing to macrosomia in women with GDM, including diet, lifestyle factors and family/personal history of GDM-M. Future studies will include a more detailed investigation by involving a larger sample of pregnant women with GDM and a more comprehensive list of factors.

## Conclusions

The 9 indicators verified by nomogram in this analysis, including pre-pregnancy BMI, weight gain at 24 gw, parity, OGTT 2 h glucose at 24 gw, HDL and LDL at 24 gw, and plasma expression of CLUL1, VCAN and RNASE3 at 24 gw, are very meaningful in terms of identifying macrosomia risk in GDM. Also, these indicators are helpful for early screening of macrosomia and the timely prevention of related complications. As a result, introducing these 9 indicators in the risk nomogram is useful for the prediction of macrosomia risk in women with GDM.

## Supplementary Information

**Additional file 1: Table S1.** The 92 Olink metabolism biomarkers showing *P* values and adjusted P value (FDR) between GDM-M and GDM-N.

**Additional file 2: Table S2.** The raw (NPX) data for the Olink Proseek multiplex metabolism assay.

## Data Availability

All datasets generated for this study are included in the manuscript.
