# Supplementary material for: Maternal circulating metabolic biomarkers and their prediction performance for gestational diabetes mellitus related macrosomia
Source: BMC Pregnancy Childbirth. 2023 Feb 14;23:113. doi: 10.1186/s12884-023-05440-9 (PMC9926775; doi:10.1186/s12884-023-05440-9)
Supplement: Supplementary file 1 — Additional file 1: Table S1. The 92 Olink metabolism biomarkers showing P values and adjusted P value (FDR) between GDM-M and GDM-N. [file 12884_2023_5440_MOESM1_ESM.docx]

| **Table S1. The 92 Olink metabolism biomarkers showing *P* values and adjusted P value (FDR) between GDM-M and GDM-N** | | | | | | |
| --- | --- | --- | --- | --- | --- | --- |
| **Markers** | **Names** | **GDM-M_Mean** | **GDM-N_Mean** | **Different_NPX** | ***P* value** | **Adjusted *P* value (FDR)** |
| CLUL1 | Clusterin-like protein 1 | 3.043 | 3.559 | -0.516 | 0.000 | 0.015 |
| VCAN | Versican core protein | 1.699 | 1.948 | -0.249 | 0.000 | 0.009 |
| FCRL1 | Fc receptor-like protein 1 | 3.278 | 3.630 | -0.352 | 0.001 | 0.016 |
| RNASE3 | Eosinophil cationic protein | 4.287 | 5.206 | -0.919 | 0.002 | 0.048 |
| APLP1 | Amyloid-like protein 1 | 3.439 | 3.773 | -0.334 | 0.005 | 0.081 |
| REG4 | Regenerating islet-derived protein 4 | 6.846 | 7.139 | -0.292 | 0.006 | 0.094 |
| CANT1 | Soluble calcium-activated nucleotidase 1 | 3.779 | 3.943 | -0.164 | 0.011 | 0.143 |
| FKBP4 | Peptidyl-prolyl cis-trans isomerase FKBP4 | 0.586 | 0.856 | -0.270 | 0.013 | 0.138 |
| GHRL | Appetite-regulating hormone | 1.290 | 1.572 | -0.282 | 0.029 | 0.281 |
| CLMP | CXADR-like membrane protein | 1.082 | 0.919 | 0.163 | 0.029 | 0.256 |
| MEP1B | Meprin A subunit beta | 1.807 | 2.086 | -0.279 | 0.034 | 0.273 |
| CD79B | B-cell antigen receptor complex-associated protein beta chain | 1.196 | 1.341 | -0.145 | 0.046 | 0.334 |
| ITGB7 | Integrin beta-7 | 0.218 | 0.289 | -0.070 | 0.064 | 0.436 |
| FBP1 | Fructose-1,6-bisphosphatase 1 | 0.427 | 0.239 | 0.188 | 0.073 | 0.458 |
| CD164 | Sialomucin core protein 24 | 0.624 | 0.712 | -0.088 | 0.085 | 0.496 |
| CD2AP | CD2-associated protein | 5.694 | 5.925 | -0.230 | 0.093 | 0.512 |
| SDC4 | Syndecan-4 | 3.971 | 4.143 | -0.172 | 0.123 | 0.636 |
| CTSO | Cathepsin O | 1.142 | 1.239 | -0.097 | 0.123 | 0.602 |
| CLSTN2 | Calsyntenin-2 | 1.885 | 1.974 | -0.089 | 0.144 | 0.665 |
| ALDH1A1 | Retinal dehydrogenase 1 | 3.006 | 3.174 | -0.168 | 0.144 | 0.635 |
| MCFD2 | Multiple coagulation factor deficiency protein 2 | 1.026 | 1.117 | -0.091 | 0.146 | 0.610 |
| NPDC1 | Neural proliferation differentiation and control protein 1 | 4.295 | 4.380 | -0.085 | 0.176 | 0.704 |
| THOP1 | Thimet oligopeptidase | 3.811 | 3.916 | -0.104 | 0.189 | 0.725 |
| NPTXR | Neuronal pentraxin receptor | 3.018 | 2.798 | 0.220 | 0.193 | 0.708 |
| CTSH | Pro-cathepsin H | -0.077 | 0.049 | -0.126 | 0.195 | 0.688 |
| ENO2 | Gamma-enolase | 1.985 | 2.102 | -0.117 | 0.197 | 0.665 |
| TINAGL1 | Tubulointerstitial nephritis antigen-like | 4.125 | 4.229 | -0.104 | 0.225 | 0.735 |
| SSC4D | Scavenger receptor cysteine-rich domain-containing group B protein | 2.368 | 2.512 | -0.144 | 0.226 | 0.711 |
| CDHR5 | Cadherin-related family member 5 | 3.138 | 3.239 | -0.102 | 0.248 | 0.754 |
| CD1C | T-cell surface glycoprotein CD1c | 3.766 | 3.851 | -0.085 | 0.252 | 0.738 |
| SUMF2 | Sulfatase-modifying factor 2 | 2.100 | 2.226 | -0.126 | 0.270 | 0.766 |
| LILRA5 | Leukocyte immunoglobulin-like receptor subfamily A member 5 | 3.916 | 4.014 | -0.098 | 0.288 | 0.791 |
| SOST | Sclerostin | 2.567 | 2.651 | -0.083 | 0.301 | 0.804 |
| ADGRE2 | Adhesion G protein-coupled receptor E2 | 4.326 | 4.416 | -0.090 | 0.303 | 0.784 |
| ENPP7 | Ectonucleotide pyrophosphatase/phosphodiesterase family member 7 | 4.124 | 4.304 | -0.180 | 0.308 | 0.775 |
| CLEC5A | C-type lectin domain family 5 member A | 2.779 | 2.867 | -0.087 | 0.337 | 0.825 |
| ANGPT2 | Angiopoietin-2 | 4.476 | 4.639 | -0.163 | 0.343 | 0.815 |
| DDC | Aromatic-L-amino-acid decarboxylase | 4.734 | 4.861 | -0.128 | 0.384 | 0.889 |
| TXNDC5 | Thioredoxin domain-containing protein 5 | 0.650 | 0.711 | -0.061 | 0.399 | 0.900 |
| BAG6 | Large proline-rich protein BAG6 | 1.205 | 1.262 | -0.057 | 0.426 | 0.937 |
| ANGPTL7 | Angiopoietin-related protein 7 | 0.727 | 0.656 | 0.071 | 0.428 | 0.919 |
| ADGRG2 | Adhesion G-protein coupled receptor G2 | 0.907 | 0.953 | -0.047 | 0.439 | 0.919 |
| PILRB | Paired immunoglobulin-like type 2 receptor beta | 4.821 | 4.704 | 0.118 | 0.451 | 0.922 |
| DPP7 | Dipeptidyl peptidase 2 | -0.438 | -0.346 | -0.092 | 0.455 | 0.909 |
| METRNL | Meteorin-like protein | 2.050 | 2.088 | -0.038 | 0.473 | 0.925 |
| NTproBNP | N-terminal prohormone brain natriuretic peptide | 3.104 | 3.219 | -0.116 | 0.483 | 0.924 |
| ACP6 | Lysophosphatidic acid phosphatase type 6 | 2.005 | 2.065 | -0.061 | 0.493 | 0.924 |
| ANGPTL1 | Angiopoietin-related protein 1 | 1.848 | 1.886 | -0.039 | 0.531 | 0.974 |
| CHRDL2 | Chordin-like protein 2 | 2.380 | 2.443 | -0.063 | 0.532 | 0.956 |
| COMT | Catechol O-methyltransferase | 0.934 | 1.020 | -0.086 | 0.560 | 0.985 |
| GLRX | Glutaredoxin-1 | -0.395 | -0.312 | -0.083 | 0.563 | 0.971 |
| GRAP2 | GRB2-related adapter protein 2 | 0.923 | 0.996 | -0.073 | 0.579 | 0.980 |
| ENTPD5 | Ectonucleoside triphosphate diphosphohydrolase 5 | 0.913 | 0.949 | -0.036 | 0.589 | 0.978 |
| SERPINB8 | Serpin B8 | -1.068 | -1.172 | 0.104 | 0.590 | 0.961 |
| KLK10 | Kallikrein-10 | -3.032 | -2.989 | -0.043 | 0.590 | 0.944 |
| CDH2 | Cadherin-2 | 1.814 | 1.849 | -0.035 | 0.601 | 0.944 |
| QDPR | Dihydropteridine reductase | 2.940 | 2.993 | -0.054 | 0.615 | 0.949 |
| IGFBPL1 | Insulin-like growth factor-binding protein-like 1 | 2.284 | 2.235 | 0.049 | 0.618 | 0.938 |
| CCDC80 | Coiled-coil domain-containing protein 80 | 3.931 | 3.892 | 0.038 | 0.657 | 0.979 |
| CRKL | Crk-like protein | 4.473 | 4.542 | -0.070 | 0.715 | 1.049 |
| TYRO3 | Tyrosine-protein kinase receptor TYRO3 | 1.746 | 1.769 | -0.023 | 0.716 | 1.033 |
| ARG1 | Arginase-1 | 0.782 | 0.851 | -0.069 | 0.721 | 1.023 |
| GAL | Galanin peptides | 4.945 | 4.998 | -0.053 | 0.738 | 1.031 |
| ANXA11 | Annexin A11 | -0.013 | 0.090 | -0.103 | 0.740 | 1.017 |
| APEX1 | DNA-(apurinic or apyrimidinic site) lyase | 3.394 | 3.493 | -0.099 | 0.741 | 1.003 |
| SIGLEC7 | Sialic acid-binding Ig-like lectin 7 | 2.933 | 2.908 | 0.025 | 0.748 | 0.998 |
| FAM3C | Protein FAM3C | 5.614 | 5.590 | 0.024 | 0.752 | 0.987 |
| PAG1 | Phosphoprotein associated with glycosphingolipid-enriched microdomains 1 | 3.610 | 3.583 | 0.026 | 0.773 | 1.000 |
| NQO2 | Ribosyldihydronicotinamide dehydrogenase (quinone) | 0.173 | 0.118 | 0.055 | 0.782 | 0.997 |
| SERPINB6 | Serpin B6 | -0.810 | -0.842 | 0.032 | 0.788 | 0.991 |
| KYAT1 | Kynurenine--oxoglutarate transaminase 1 | 5.166 | 5.201 | -0.035 | 0.801 | 0.993 |
| LRIG1 | Leucine-rich repeats and immunoglobulin-like domains protein 1 | 3.282 | 3.265 | 0.017 | 0.818 | 1.000 |
| ANXA4 | Annexin A4 | -0.458 | -0.490 | 0.032 | 0.826 | 0.996 |
| USP8 | Ubiquitin carboxyl-terminal hydrolase 8 | 0.944 | 0.923 | 0.021 | 0.827 | 0.983 |
| NOMO1 | Nodal modulator 1 | 5.433 | 5.446 | -0.013 | 0.841 | 0.987 |
| S100P | Protein S100-P | -1.675 | -1.710 | 0.035 | 0.842 | 0.975 |
| CA13 | Carbonic anhydrase 13 | 1.036 | 1.063 | -0.026 | 0.861 | 0.983 |
| HDGF | Hepatoma-derived growth factor | 0.552 | 0.529 | 0.023 | 0.915 | 1.033 |
| ROR1 | Inactive tyrosine-protein kinase transmembrane receptor ROR1 | 3.717 | 3.712 | 0.006 | 0.941 | 1.049 |
| TYMP | Thymidine phosphorylase | -1.038 | -1.047 | 0.009 | 0.942 | 1.036 |
| LRP11 | Low-density lipoprotein receptor-related protein 11 | 3.940 | 3.946 | -0.006 | 0.943 | 1.025 |
| SEMA3F | Semaphorin-3F | 2.779 | 2.775 | 0.005 | 0.947 | 1.016 |
| RTN4R | Reticulon-4 receptor | 1.388 | 1.383 | 0.005 | 0.950 | 1.007 |
| TFF2 | Trefoil factor 2 | 5.548 | 5.535 | 0.013 | 0.954 | 0.999 |
| NADK | NAD kinase | 4.195 | 4.181 | 0.014 | 0.956 | 0.989 |
| NECTIN2 | Nectin-2 | 5.024 | 5.020 | 0.004 | 0.957 | 0.979 |
| SNAP23 | Synaptosomal-associated protein 23 | 2.195 | 2.202 | -0.007 | 0.966 | 0.977 |
| PPP1R2 | Protein phosphatase inhibitor 2 | 2.099 | 2.095 | 0.004 | 0.985 | 0.985 |
| AHCY | Adenosylhomocysteinase | N/A | N/A |  |  |  |
| DAB2 | Disabled homolog 2 | N/A | N/A |  |  |  |
| DIABLO | Diablo homolog, mitochondrial | N/A | N/A |  |  |  |
| TSHB | Thyrotropin subunit beta | N/A | N/A |  |  |  |
| N/A, Not available; FDR, False positive rate; GDM-M, women with gestational diabetes mellitus and macrosomia; GDM-N, women with gestational diabetes mellitus and normal neonatal weight. | | | | | | |
